# What a critical public health perspective can add to the analysis of healthcare responses to gender-based violence that focus on asking

**DOI:** 10.1186/s12889-023-16641-4

**Published:** 2023-09-06

**Authors:** Isabel Goicolea

**Affiliations:** Department of Epidemiology and Global Health, Umeå University, Umeå, Sweden

**Keywords:** Gender-based violence, Asking, Public health, Health-care

## Abstract

In this comment I analyze the effects of approaching gender-based violence as a public health problem, that the health system should address through ‘daring to ask’. I acknowledge the potential of the ‘daring to ask’ strategy, but I also argue that asking has effects, and that we should be aware of them.

## Introduction

Gender-based violence (GBV), especially intimate partner violence against women, is increasingly represented as a public health problem [1–4]. As a consequence of this, the healthcare system’s responsibility towards women victims/survivors^1^ of GBV has become visible within both national and international policies [5–7]. Central to the healthcare system’s response to GBV is the identification of violence committed against women users of healthcare services, mainly through ‘daring to ask’ them about GBV in more or less standardized ways [3, 8–10].

The discipline of public health has contributed to better understand and address GBV through, for example extensive and important research investigating screening and other approaches to asking, as well as research exploring the challenges and opportunities involved in identifying GBV within healthcare services (to mention just a few examples [8, 9, 11–14].

What is lacking, however, is a critical examination of the material and discursive effects of addressing GBV as a public health problem, which the healthcare system should address through daring to ask. A critical public health perspective, that problematizes new normativities, challenges taken for granted assumptions, analyses unintended effects of public health interventions and looks for ways to address them, provides a fruitful approach to engage in such examination [15, 16] With this commentary, my purpose is to start to open up such an examination and to argue for why it is needed. In doing this I am guided by four research questions:

i) Is GBV a public health problem? ii) Should the healthcare system do something about it? iii) Is ‘daring to ask’ the way in which the healthcare system should respond to GBV? And the central, cross-cutting, question: iv) What are the effects of approaching GBV as a public health problem, that the healthcare system should address through daring to ask?

Taking a critical public health approach, I build upon my own research on the healthcare system’s response to GBV in Spain, a research project in which we analyse ‘daring to ask’ in the work of social services in Sweden, as well as seminal work on public health, gender-based violence, health and healthcare, to provide, not definitive solutions but, provisional and partial answers that may open up for further questioning. Such a contribution is relevant conceptually and theoretically and its transferability is not limited to one specifical geographical context, because it builds upon not only empirical research but also theoretical work. Concepts and names are important and complex when it comes to the field of gendered violence (see Boyle [17] and Frazer & Hutchings [18] for comprehensive reflections on the topic). In this commentary I chose to use the concept gender based violence (GBV) because I consider it bread enough to cover many of the types of violence I refer to, and because it focuses on the reasons/roots of violence (gendered relations and structures). However, I acknowledge that most of the studies and protocols related to the health system response address mainly intimate partner violence and sexual violence against women, which are an important part but not the only types of violence within the broader spectrum of GBV.

### Is GBV a public health problem? What are the effects of approaching GBV as a public health problem?

In public health, we focus on problems that affect a large proportion of the population and/or are unequally distributed. We are especially interested in analysing how social factors (such as class, racialization, disability, or gender) influence health and access to support [15, 16]. Public health is highly political and visionary. In the words of Frances Baum, the new public health aims not only to improve the health of the population, but also to make the world more fair and just [15].

From these standpoints, GBV qualifies as a public health problem. Firstly, it is common and pervasive. Across her lifetime, one in every three women, or around 736 million women worldwide will be subjected to physical or sexual violence by an intimate partner or sexual violence from a non-partner, and this number has remained quite stable during the last 10 years [19, 20]. Secondly, GBV damages health and wellbeing. It is associated with a broad range of health effects; to name just a few, it increases the risk of sexual and reproductive health problems, mental health problems, chronic diseases and death. Furthermore, the harmful consequences of violence continue over time, even after the violence ends [3, 20–23]. Thirdly, GBV is unequally distributed, unfair and unjust. Intimate partner violence affects women more severely than men, and feminist theorizing has provided enormous amounts of evidence supporting that patriarchy, sexism and gender inequities are at the root and perpetuates GBV [17, 18, 14–26]. In understanding the pervasiveness of GBV it is crucial to consider the intersection of gender with other axes of oppression; trans women, women with disabilities, migrant women, younger women, and racialized women are all at higher risk of violence, and face more barriers to accessing support [3, 19].

Despite its high prevalence and harmful health consequences, GBV was not conceptualized as a public health problem until the 1980s. Prior to that, it was understood primarily as a social or legal problem, or even a private one [1]. Changes in the conceptualization and visibilization of GBV as a public problem did not happen spontaneously. It was the result of the fight of feminist organizations and the shelter movement that pioneered mobilizing against GBV, both by providing individual support to victims and positioning GBV as a gendered problem [27].

Nowadays, approaching GBV as a public health problem permeates the ways in which we talk about it. For example, it is very common to use epidemiological language to highlight the extent, relevance and urgency of the problem of GBV [28] GBV is described as an epidemic, or, more recently, a pandemic. This language presents GBV as a problem similar to other health problems that are widespread globally. If GBV is an epidemic, it is no longer understood as anecdotal or rare, but as common, embedded within society, and an urgent problem that needs to be tackled.

From a public health perspective, GBV is also presented as a risk factor that can be screened for. Since it is linked with several health problems, and the harmful effects continue long after the violence has ended, detecting GBV becomes a prioritized arena for preventing potential negative health effects. While this can be absolutely helpful, Sweet warns of the risk of determinism (for women subjected to GBV, the future is imagined as already in jeopardy), of diagnostic bias (the risk of explaining all the health problems of women subjected to GBV as linked to violence), and biomedical surveillance of victims [4, 29, 30].

In sum, the conceptualization of GBV as a public health problems has helped to make GBV visible and has highlighted its pervasiveness, harmful effects and the urgency to respond to it within the health system. However, it also brings the risk of determinism and increased control of victims/survivors and of turning a blind eye on the perpetrators of GBV and the structural factors perpetuating it.

### Should the healthcare system do something about it? What are the effects of addressing GBV as a healthcare problem?

GBV is an important risk factor for a range of health problems, and research shows that women who are subjected to GBV use the healthcare system more than those who are not [31]. In addition, healthcare services are the public services most frequently used by women subjected to GBV, more than legal support, social services, or the police. Healthcare services then become a door opener for identifying violence, providing support, and referring victims to other services. Despite these strong arguments as to why the healthcare system should become involved in addressing GBV, this violence is seldom spontaneously discussed during encounters with healthcare professionals [3, 8, 14]. This, as I discuss below, is the argument for asking about GBV.

Both the CEDAW and the Istanbul Convention (which are the most influential international treaties addressing GBV) include the healthcare sector as an important actor [5, 7], and there are World Health Organization (WHO) guidelines describing the six key components of the health sector’s response: women-centred care, identification and care of victims, clinical care for sexual violence, training of healthcare professionals, policies and guidelines, and the importance of respecting women’s wishes instead of pushing for compulsory reporting [3]. While guidelines have been instrumental both in positioning GBV in the public (health) agenda and in providing guidance to health care professionals, there has been criticism in relation to some aspects (for example, how to provide women-centred care in practice), and that implementation remains erratic and highly dependent on the interest and commitment of individual professionals [2, 10].

Addressing GBV within the healthcare system has effects. On the one hand, it offers an opportunity to detect and refer a problem that may otherwise remain unnoticed and unaddressed [8]. Since using healthcare may be less stigmatized than using other types of welfare services (like social services), it can contribute to reducing the stigma still attached to being a victim/survivor of GBV. At a collective level, it sends the message that addressing GBV is a public/state responsibility and not a private matter. In addition, integrating the response to a complex problem rooted in gender inequities such as GBV within the healthcare system can also contribute to broadening the healthcare system’s perspective on health and ill health, in relation to the causes of illness [32], and especially in relation to gender as a crucial social determinant of health. All these effects are undoubtedly beneficial for victim/survivors and have the potential to challenge society’s perception of GBV, and (if I am allowed to be optimistic) can contribute to make health care systems more holistic.

On the other hand, some of the potential effects of addressing GBV within the healthcare system are more problematic. Healthcare responses still focus on providing a solution for each individual user, which means that the social aspects of the problem become downplayed. The gendered aspects of GBV can become invisible when responses to it take place only at an individual level and focus on documenting and curing the trauma and injuries experienced by each user [2, 4, 29]. The core of the problem, that GBV is rooted in gender inequities and patriarchy, remains unchallenged.

Addressing GBV within the healthcare system also represents a shift in who the expert is considered to be: from women to (healthcare) professionals. For example, the healthcare system is increasingly involved in granting legitimacy to victims of GBV, for example through forensic reports or through the documentation of abuse within clinical records. In countries such as Spain, while it is no longer required for a woman to make a legal complaint to access resources, she does need to have the ‘stamp’ from certain healthcare professionals to be considered as a GBV victim, and consequently become able to access support [33]. The healthcare system then becomes not only a door opener for resources but also a powerful gatekeeper.

Finally, responding to GBV by means of the healthcare system also means that certain forms of violence and certain responses which are more familiar to this system are more easily implemented, while other become disregarded; for example, treating physical injuries, providing clinical care for survivors of sexual abuse, or identifying GBV through asking are responses that the health care system is familiarized in providing. Certain forms of GBV (for example, psychological violence, coercive control) are less easy to recognize, and other types of response (promotional work, psychological support) are less easy to implement. What is complicated to address within the health care system remains then unaddressed, which also contributes to its invisibilization. Victims/survivors’ needs are responded to, at best, partially.

### Is ‘daring to ask’ the way in which the healthcare system should respond to GBV? What are the effects of focusing healthcare responses to GBV on ‘daring to ask’?

A core strategy for how the healthcare system addresses GBV is to support women who come to healthcare facilities to disclose GBV. This means that professionals identify the violence, mainly through ‘daring to ask’ all women, or women ‘at increased risk’, or those women whom the professionals suspect may have been subjected to violence. The underlying assumption seems straightforward: if professionals become aware that a woman they are meeting has been subjected to GBV, they will be able to offer her support, either directly or through referring her to appropriate resources [33].

In many countries, asking about GBV is central to healthcare strategies for addressing violence [9]. For example, in Spain, it is included within the national and regional protocols [10], and in Sweden, ‘to dare to ask’, is recommended within the policies and guidelines that govern the work of healthcare services [2].

The strategy of addressing GBV within healthcare services through ‘daring to ask’ has effects. At an individual level, it can support the disclosure of difficult events and, if done with a ‘proper’ attitude, can contribute to sanctioning women’s experiences [34]. It can be the first step to opening up access to certain services. Asking can support women in ‘becoming aware’ that what they are experiencing is not ‘normal’ or acceptable, and that there is a label that can be used to speak about it [33]. Through being asked, women receive the message that they can access support from professionals, now or in the future.

At a collective level, asking is part of making visible public responsibility for addressing GBV [9]. It makes it easier to send a concrete and specific message about what is expected from public institutions and how they can be held accountable: ‘daring to ask’ is both a powerful and a simple message. And, finally, programmes that focus on asking *all* women contribute to gendering violence and reducing the stigma attached to being a ‘victim’ – violence is something that could happen to any woman, simply because she is a woman.

However, there are also other effects that it is important to problematize. Disclosing GBV comes with consequences and expectations, not only for the professionals who provide support, but also for women. Carbin, for example, highlights how, rather than merely being a way to relieve stress and gain access to services, disclosure can become a responsibility and a requirement for receiving support [35]. Disclosing GBV comes with the expectation that women will do something about it; namely, that they will leave the abusive relationship. This could be an explanation for why disclosure frequently does not precede action, but rather, the action comes first. Or, as Enander describes it: ‘‘women do not leave because they realise they are abused; rather, they realise they are abused because they have left” [36], p.218). Under such circumstances, disclosure may not always be a door opener or first step.

When it comes to asking as a way to support disclosure, research shows that women are more likely to disclose as part of an ongoing conversation with relatives and friends than as the result of a unidirectional asking and telling with a professional [33, 37]. And while I do not mean that healthcare professionals’ approach consists of mechanically asking and telling, the limited time available during healthcare consultations may mean that asking is undertaken in a more directed, structured, and standardized way than during a conversation. Asking in a standardized manner might produce a ‘one size fits all’ approach that may prove problematic, because the needs of different women differ.

There may be challenges to reconciling an approach that considers all women ‘at risk’ ‘because of being women’ [18], while simultaneously recognizing the special situations of vulnerability and needs that a victim of violence may have due, for example, to her age, class, or racialization. Asking all women is also a ‘double-edged sword’. It may leverage stigma and contribute to the representation of GBV as being grounded in gender inequities, but it can also contribute to the representation of women as a vulnerable group, place the responsibility on individual women, and downplay the responsibility of both the men perpetrating abuse and the societal norms perpetuating GBV.

And, finally, just as important as asking the questions, is how those questions are asked. Firstly, this is because asking in a judgemental way instead of supporting disclosure and opening up access to resources may have the completely opposite effect – women may feel ashamed and re-interpret their experiences as being a ‘normal part of women’s life’ [34]. Secondly, there is a risk that too much hope will be invested in disclosing as the solution [2]. Too much focus on disclosure risks positioning it as a measure of success, or an end in itself, disregarding the reality that after disclosing women still face many challenges and have diverse needs that still have to be addressed [33].

## Conclusions: why a critical public health perspective is needed

In summary, GBV is a public health problem, but it is also a social, gendered, and racialized problem. The healthcare system can play a role in addressing the needs of women subjected to GBV, but this must necessarily be in coordination with other sectors. Representing GBV as a public health problem, which the healthcare system should address, opens up certain possibilities, but it may also close down others. The centrality of the strategy of daring to ask can be seen as a consequence of such representations: if GBV is a healthcare problem that should be dealt with within healthcare, it becomes easier to do so through strategies that are familiar to this system, such as asking and identifying.

So, what are the consequences of this? If ‘daring to ask’ becomes a somewhat problematic strategy, are there other, alternative models for responding to GBV within healthcare?

I do not *dare* to answer this. What I dare to argue here is that asking has effects, and that we should be aware of them and try to mitigate those that may be harmful, or even dare to imagine other ways to respond to the needs of women subjected to GBV who come to healthcare services.

This is not a trivial question, or merely a rhetorical exercise. Problematizing new normativities, challenging taken-for-granted assumptions, analysing the unintended effects of public health interventions and looking for ways to address them is at the core of critical public health [16]. I am aware that, in the current political context, such critical perspectives can be instrumentalized by groups that oppose progress in policies and strategies to prevent GBV and address victims’ needs. We need to be fierce, clear, and careful, because such perspectives are needed now more than ever.

From such a perspective, we can suggest, for example, that disclosure of GBV should be done in a way that ensures that, when women disclose GBV to healthcare professionals, their experience extends beyond asking and answering, into an empathic conversation during which they feel validated. And healthcare professionals should have the time, skills, and support to accomplish that. We also suggest that it is urgent to find ways to leverage the responsibility and expectations placed on women who disclose, which include to do something, to leave the abusive partner, to file a denunciation. It is also important to promote other ways of legitimating women’s experiences of GBV without the requirement for a professional’s endorsement. And it is crucial that disclosure does not become a requirement, but rather is just one possibility, and that other ways of addressing GBV beyond disclosure are also explored. Disclosure cannot become the marker of success per se, without further follow up of what happens after disclosure. Finally, we suggest that individual-based solutions around supporting disclosure are important responses but they are not enough. Consequently, collective feminist spaces and political mobilization are needed to problematize GBV and challenge societal perceptions of such violence. Now, more than ever.

## Data Availability

Not applicable.

## Footnotes

1 By using victim/survivor I want to acknowledge that the experiences of women exposed to GBV are diverse and fluid. I do not want to label them as victims (only), since the concept can be read as disempowering and paternalistic. However, the term survivor can also be problematic, since it neglects the experience of those women who are killed, for example. In addition, it brings with it a responsibilization of women exposed to GBV to overcome, move from the position of victim to that of survivor, which can be also understood as establishing hierarchies between more desirable (survivors) and less desirable (victims) subject positions. Studies and reports of women exposed to GBV show that experiences are diverse, fluid, and that women may feel both as victims and survivors at the same time.
